# THE HARTLEY GUINEA PIG: A NOVEL MODEL FOR PROGRESSIVE NEURODEGENERATION?

**DOI:** 10.1093/geroni/igae098.2442

**Published:** 2024-12-31

**Authors:** Kristen Glennie, Amanda Latham, Arsha Moorthy, Maureen Walsh, Thomas LaRocca, Kelly Santangelo, Julie Moreno, Karyn Hamilton

**Affiliations:** Colorado State University, Fort Collins, Colorado, United States; Colorado State University, Fort Collins, Colorado, United States; Colorado State University, Fort Collins, Colorado, United States; Colorado State University, Fort Collins, Colorado, United States; Colorado State University, Fort Collins, Colorado, United States; Colorado State University, Fort Collins, Colorado, United States; Colorado State University, Fort Collins, Colorado, United States; Colorado State University, Fort Collins, Colorado, United States

## Abstract

Alzheimer’s Disease and Alzheimer’s Disease Related Dementias (AD/ADRD) affect an estimated 55 million people worldwide; a staggering figure that is expected to grow in the coming years. With these projections looming, we have yet to identify any cures, treatments, or preventative strategies. Historically, AD/ADRD research is conducted using a genetically engineered model, that expresses a specific brain aging pathology. Modern research, however, has identified a complex and dynamic “inflammaging” phenotype that exists with and contributes to AD/ADRD pathology. Currently, we do not have an accessible or tractable preclinical model, that naturally mimics age-related progressive neurodegeneration in humans. Recent findings, however, suggest the Hartley guinea pig may address this need. Hartleys develop systemic inflammation and progressive age-related comorbidities characteristic of human aging. This whole-body aging phenotype prompted analyses in the brain, which found Hartleys exhibit sequence homology and similar transcriptomic mechanisms of human brain aging and AD. Hartleys also develop evidence of neuroinflammation and neurodegeneration in the hippocampus. To further investigate these findings, we examined the histopathology of 4 brain regions often implicated in neurodegenerative decline, for evidence of progressive neuropathology. Analysis is ongoing, though early results support the presence of a neuroinflammatory age-related phenotype. Additionally, biomarker variability between each Hartley may mimic the natural progression of human ‘brain aging’; though more data are needed before conclusions are drawn. Findings from this study will contribute to the hypothesis that AD/ADRD is a whole-body disease, and ultimately support the goal of closing the existing translational gap between preclinical and clinical neurodegenerative research.

